# Remote Musculoskeletal Assessment Framework: A Guide for Primary Care

**DOI:** 10.7759/cureus.12778

**Published:** 2021-01-19

**Authors:** Tom Murray, Gemma Murray, James Murray

**Affiliations:** 1 Medicine, Clifton College, Bristol, GBR; 2 Students' Health Service, University of Bristol, Bristol, GBR; 3 Avon Orthopaedic Centre, Southmead Hospital, North Bristol NHS Trust, Bristol, GBR; 4 School of Medicine, University of Bristol, Bristol, GBR

**Keywords:** remote consultation, primary care medicine, musculoskeletal assessment, remote msk examination, video telemedicine, e-consult, telemedicine, orthopaedics, primary care education

## Abstract

Introduction

Remote consulting has exploded into primary care following the initial COVID-19 surge as a measure to reduce potential cross-infection (staff-patient or patient-patient). Musculoskeletal (MSK) conditions comprise up to 21% of the annual primary care caseload in England. Established techniques for MSK examination, however, rely on face-to-face attendance. Evidence-based guidance for remote MSK assessment is required to ensure the quality of care is maintained with the move from face-to-face to virtual consultations.

Method

A literature review of published evidence and current guidelines was conducted. The most appropriate remote consultation techniques and MSK examinations were identified and where there was no evidence, modified examination tests were developed from established face-to-face examination techniques. A concise, accessible framework for remote MSK assessment in primary care was then created and tested on a non-medically trained volunteer.

Results

Over 2232 papers and articles were identified by search headings, reducing to 28 sources that had relevant content. At the time of searching, there was no published evidence relating to MSK remote consultation in a primary care setting. However, evidence was found in the physiotherapy and rehabilitation literature for the efficacy and practicality of MSK teleconsultation.

MSK remote examination framework

From this literature and with the addition of modified established examinations, an MSK assessment framework was constructed. This framework provides pre-consultation guidance and step-by-step remote examination instructions. Patient and clinician resources (including a patient information leaflet and photographic examples of examinations) were created as supplementary material.

Conclusion

Due to the frameshift away from face-to-face consultation, primary care clinicians have found themselves lacking an evidence base or practical guidance to support remote MSK assessment. This paper is a systematic literature review of MSK telemedicine from which practical advice and evidence-based MSK tests have been developed. Where there is no evidence, modified traditional tests are suggested to allow a complete framework for remote MSK examination - using a system approach of ‘look, point, move’ followed by modified special tests, for use in a primary care setting as a ‘ready-to-use’ practical guide to remote MSK assessment, presented in a downloadable format.

What did this add?

With 21% of primary care consultations relating to MSK conditions and limited means of performing face-to-face MSK examination due to COVID-19, there needs to be a recognised framework for assessing the MSK system remotely. To the best of our knowledge, this evidence does not exist for primary care remote MSK examination. This paper demonstrates evidence-based practical advice (from non-primary care settings) and modified MSK examinations to be used in a primary care MSK remote consultation.

## Introduction

Twenty-five percent of the UK population suffer from musculoskeletal (MSK) conditions, with a significant number of these patients being of working age [1]. These MSK conditions consist of more than 150 diagnoses of the locomotor system [2]. The Institute for Health Metrics and Evaluation in 2017 estimated that 18 million people lived with an MSK condition in the UK, which is thought to cost the NHS £5 billion each year [3,4]. Furthermore, MSK conditions account for up to 21% of annual primary care consultations across England [5].

The COVID-19 pandemic has driven demand, both from patients and providers, for the increased use of remote consultation; in the first week of March 2020, there was a 200% rise in the number of primary care telephone and video appointments across the UK [6]. NHS England recently reported that primary care consultations have moved from 95% face-to-face to over 85% remote during the COVID-19 pandemic [7].

The remote management of MSK conditions presents a number of challenges for primary healthcare, especially in conjunction with the COVID-19 pandemic meaning traditional, face-to-face physical examination has not been possible for most appointments, which were predominantly telephone consultations. The traditional model of care for MSK conditions involved a physical examination. Therefore, it is important to explore what is feasible during remote MSK examination, perhaps with certain modifications. For example, where a “GALS” (Gait, Arms, Legs and Spine) screen would normally be performed face-to-face, similar MSK screening examinations need to be adapted for remote consultation [8].

Video teleconsultation offers a possible solution, but there remains a lack of published literature on how to modify the MSK examination when working remotely. This paper demonstrates evidence-based practical advice and modified MSK examinations for a primary care teleconsultation to allow a ‘new normal’ to be established for primary care clinicians on how to approach MSK examination via teleconsultation.

Telemedicine is defined as ‘the practice of medicine via a remote, electronic interface’ and in 2019, was found to be the fastest-growing sector of health care [9,10]. This growth is associated with the benefits of the absence of travel time, reduced time of work for patients and increased patient satisfaction [11]. In 2014, Smith et al. published a study from the Royal Children’s Hospital (RCH) in Queensland, Australia which showed that telehealth appointments had reduced failure-to-attend rates, from an average of 27% (face-to-face) to 7% (remote access) [12].

Prior to the COVID-19 pandemic, the NHS long-term plan committed that by 2023, every patient in England would be able to access a digital primary care service, within which teleconsultation would be a significant factor [13].

The most common form of telemedicine in a primary care setting is teleconsultation - either through real-time video/audio calls or by ‘store and forward’ techniques [2]. Live methods involve a simple telephone call or video consultation (e.g., using platforms such as AccuRx, EMIS Health and Attend Anywhere (Appendix 1.1) or widely used platforms like Zoom, Skype, WhatsApp Video, FaceTime and Microsoft Teams). Store and forward techniques use text messaging platforms to contact and monitor patients remotely.

A number of studies have taken place to establish the validity and reliability of MSK telerehabilitation with physiotherapy of the knee, elbow, shoulder, lower back and ankle [14-18]. The remote assessment of MSK disorders by physiotherapists was studied in a systematic review [19] by Mani et al. in 2017 and found to be technically feasible, with ‘good-to-excellent’ validity and reliability. Furthermore, Cottrell et al. [20], in 2016, performed a systematic review and meta-analysis, analysing the effectiveness of real-time telerehabilitation with MSK conditions when compared to standard face-to-face practice; this review demonstrated telerehabilitation to be effective in improving physical function, disability, and pain. With regard to clinical diagnostic accuracy, there are certain areas (e.g., range of movement) where telerehabilitation is superior to face-to-face consultation [21]. Patient satisfaction has also been shown to be high in telerehabilitation, with remote consultation being ‘well-received’ by participants and even preferred over conventional methods [22,23].

As in a face-to-face consultation, the instinct of the clinician should not be disregarded. It is not yet known how this key ‘sixth sense’ will evolve into the digital age of consultation. Taking a careful history is vital and awareness of MSK ‘red flag’ conditions is still essential. These include malignancy, acute neurological loss (particularly cauda equina syndrome), bone and joint infection, inflammatory arthritis, giant cell arteritis (GCA) and acute trauma. The presence of any ‘red flag’ symptoms requires consideration for further investigation, safety-netting, referral (routine or urgent), or emergency hospital admission [24] (Appendix 1.2).

Remote assessment must not be seen as mutually exclusive from an additional face-to-face consultation in specific cases. Thus, teleconsultation could be used as a triaging or information-gathering tool to allow clinical decision making for further investigation or signposting - allowing the patient to be directed to the most appropriate clinician for a face-to-face examination.

Although there is an abundance of research in MSK telemedicine in the field of physiotherapy, to the best of our knowledge, there is a deficiency of MSK teleconsultation research and advice targeted at primary care providers. Given the time-constraint of 10- to 15-minute appointments, a succinct approach to MSK remote assessment is vital. Our aim was to produce a pragmatic, yet efficient method for patients to perform self-tests using specific teleconsultation instructions and example images to allow the ‘virtual examination’ of a patient’s MSK system remotely.

## Materials and methods

A structured search and review of the current literature in this field was performed in July 2020. Literature regarding telemedicine, MSK conditions and primary care, using both academic and non-academic search engines was identified. The search engines used were PUBMED, Google Scholar, YouTube and Google, in addition to primary care medical education resources. The search strategy used the medical subject headings (MeSH) listed in Table 1.

**Table 1:** Medical Subject Headings (MeSH)

‘Hip’
‘Knee’
‘Spine’
‘Shoulder’
‘Back’
‘Musculoskeletal’
‘Orthopaedic’
‘Primary care’
‘Remote consultation’
‘Online consultation’
‘Video consultation’
‘Teleconsultation’
‘Zoom consultation’
‘Teams consultation’
‘Telemedicine’
‘Met-analysis
‘Metanalysis’
‘Meta-analysis’
‘Systematic review’
‘Review’

**Figure 1:**
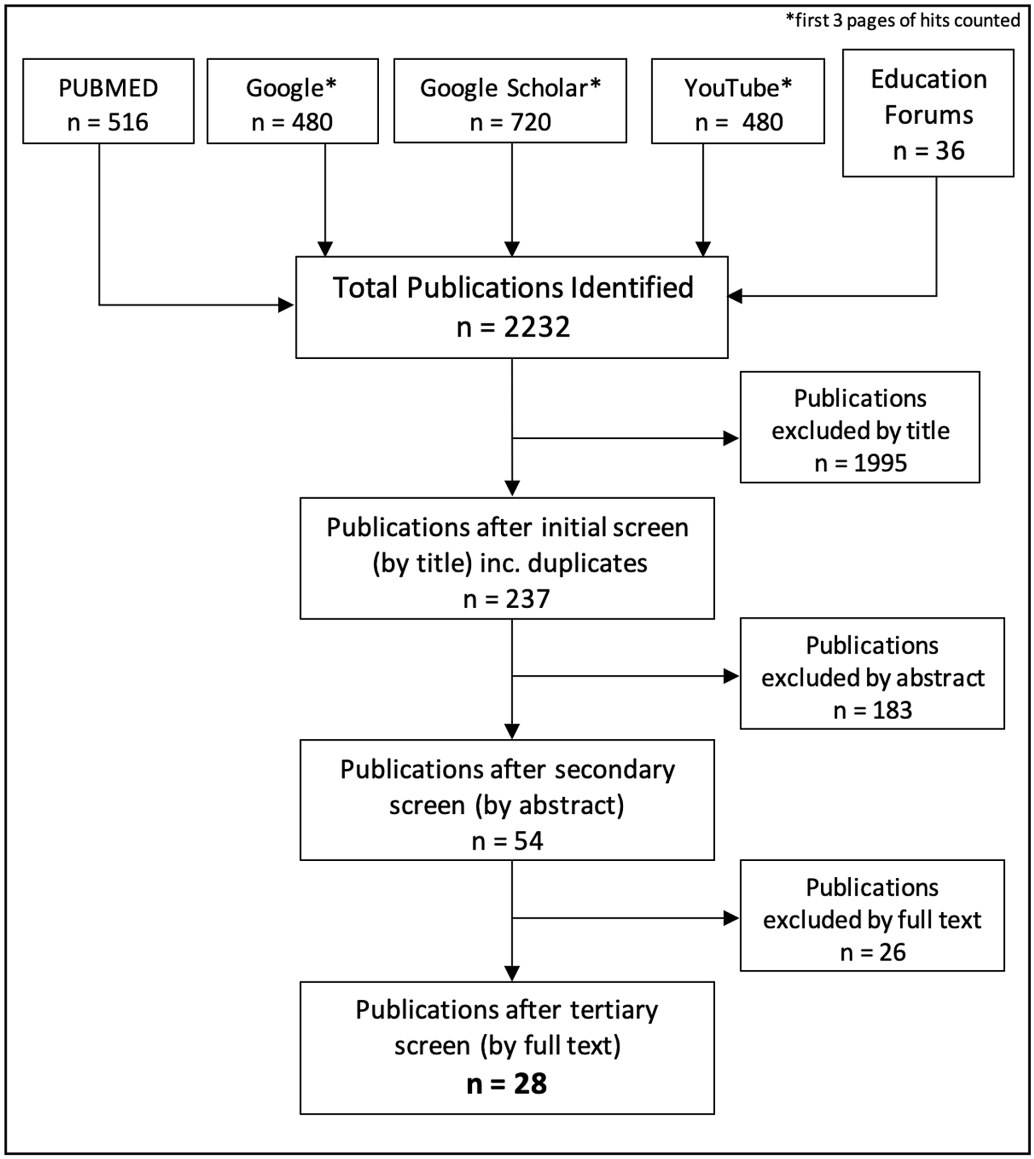
Literature Review Strategy

This produced 2232 matches which were screened as per Figure 1, initially through title and then abstract, before complete paper review for relevant publications. The remaining relevant 28 publications were used as the available evidence base at the time of the study.

A list of commonly used MSK examination tests was then devised and limited to tests applicable in a primary care setting. Where there was evidence for a particular test remotely, it was included, but when not available, modifications of face-to-face techniques were made, using the experience of a practicing GP (GM) and orthopaedic consultant (JM). Careful command sentences were then constructed to achieve clear instructions for a patient remotely. Photographs were then taken of each test.

The modified examinations were presented in a uniform way using bullet point format alongside photographic demonstrations of normal tests and abnormal tests where appropriate. These adapted examinations were tested on a non-medically trained volunteer (TM) to check the efficacy of the suggested verbal and photographic instructions.

## Results

**Figure 2:**
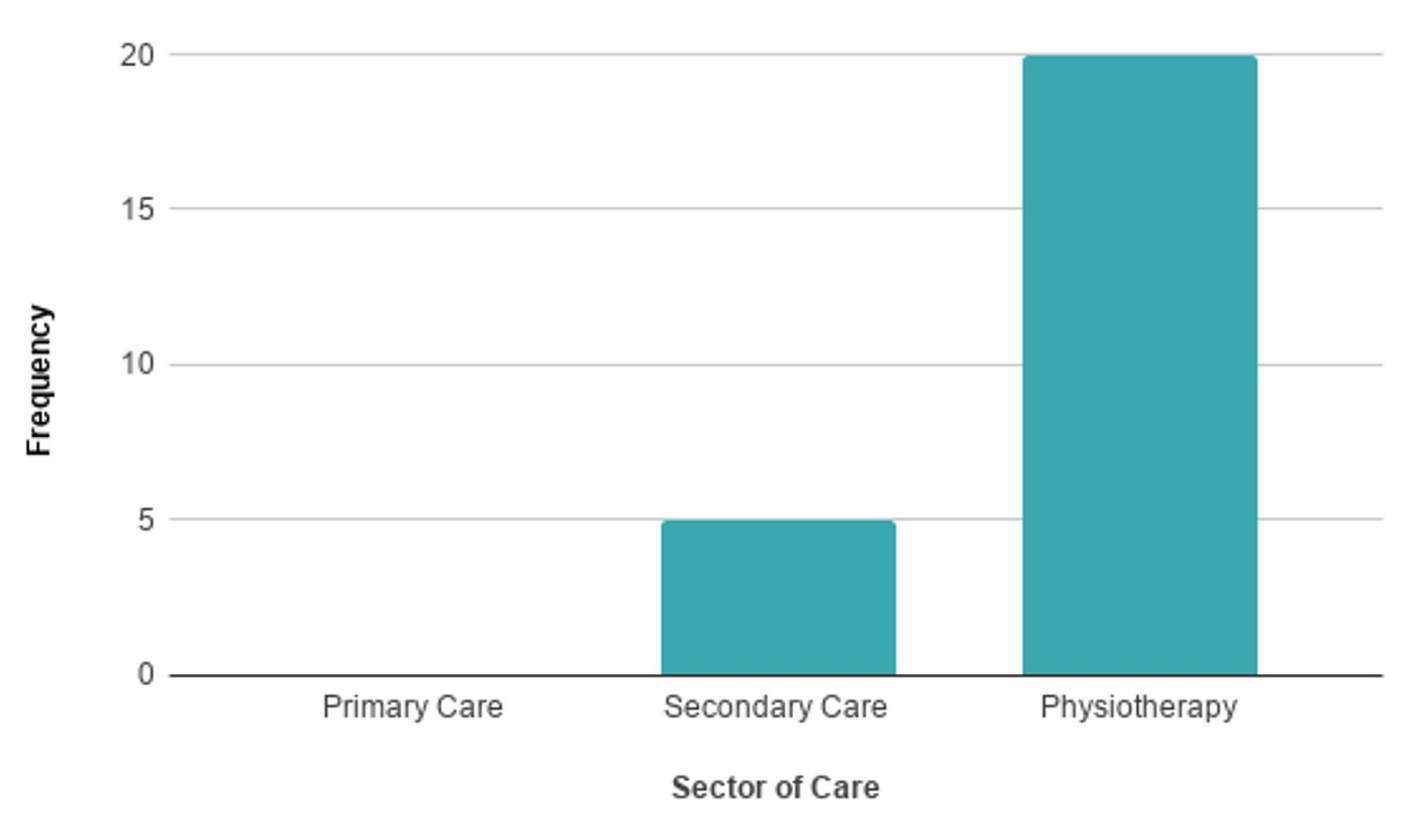
Relevant Published Literature for MSK Telemedicine MSK: musculoskeletal.

Following the literature search and review, a lack of information regarding MSK remote examination was identified. This is displayed in Figure 2 which shows the relevant published literature for MSK telemedicine within different sectors and highlights the lack thereof in primary care.

Evidence-based practical advice for MSK teleconsultation

Pre-Consultation

In advance of the remote consultation, patients should be sent any relevant consent information as per all remote consultations [25]. Where possible, it is advised that patients are provided with information before the examination regarding the practicalities of their teleconsultation including appropriate devices (laptop or tablet rather than a desktop computer or mobile phone), lighting (directed towards the patient, not the camera), location (quiet and safe for examination) and clothing (allowing appropriate joint visualisation without exposure of intimate areas) [26]. This presents a potential opportunity to discuss the requirement of a chaperone for a certain examination. It has already been proven that factors including bandwidth limitations, low camera resolution and bad lighting, have all been associated with poor validity [15]. Other suggestions include providing pre-prepared photographs or videos to demonstrate MSK tests that may be used (Appendix 1.3).

A detailed explanation of these key ideas has been summarised in Table 2. It may be useful to provide this information to patients with a similar checklist upon booking an MSK teleconsultation [26]. Potentially, this may be instigated during a phone consultation - gathering information - highlighting the need for further investigation. Thus, the patient may be sent this guidance to optimise their next remote (video) assessment when a more specific examination would be performed (demonstrative materials are illustrated in the figures).

**Table 2:** Patient Teleconsultation Advice Sheet

Patient Teleconsultation Advice Sheet
Before your appointment
Please read any information sent to you in readiness for your remote consultation. This may include details about consent, accessing your remote consultation, or how to use the video software. If your internet bandwidth is limited, you may consider asking others for priority use of the internet during your consultation or join the consultation from an alternative internet connection, e.g., a friend or relative’s house. The use of a tablet or laptop rather than a desktop computer or mobile phone is recommended to allow ease of movement and optimal screen size. Ensure that the device to be used is fully charged. Please ensure that your correct telephone number is known to the healthcare provider. If the video connection is lost, a telephone call may be required to complete the consultation.
Where should you be for the consultation?
You will need to be located in a quiet room, with adequate space for movement tests. You should be positioned with an uncluttered background and good front lighting but ensure not to position the light source to shine directly at the camera. It may be helpful for a trusted volunteer to assist with camera positioning. The movement of the camera during the consultation should be avoided unless requested by the clinician.
Spine, Shoulder, Elbow, Wrist, Hand - position the camera at dining table height. Ensure there is space to stand 6 ft (180 cm) away and to move your arms in all directions. Have a pen and key available for specific tests.
Hip, Knee, Ankle - position the camera at chair or coffee table height. Ensure there is space to stand 6 ft (180 cm) away. You may need to stand, sit, or lie on the floor with legs fully extended and to sit on a chair.
What to wear?
Shoulder, Elbow, Spine – clothing above the waist will need to be removed. Women should wear a vest/crop top or bra. Wrist and Hand – wear short sleeves and remove any wrist/hand jewellery. Hip, Knee, Ankle – wear shorts and feet should be barefoot.
What to expect on the day?
The teleconsultation will begin with a brief introduction, confirmation of identity, verification of privacy in your location and confirmation of consent for remote consultation. There will then be a discussion about your problem and any previous treatment or medication. There may be a remote examination. Visual access to the problem joint or limb is essential. Photo examples of specific tests may be displayed by the clinician using a ‘share screen’ function. The consultation will conclude with a management plan which may include treatment advice, exercises, further investigation or referral to a specialist.

Patient Environment and Attire

It is advisable that the patient is located in a quiet room of adequate size for a range of movement tests. The patient should be positioned with an uncluttered background and appropriate front lighting to avoid glare into the camera. A number of references also suggested the benefit of a trusted volunteer being available to help with camera positioning in order to maximise consultation efficiency by positioning the device (ideally laptop or tablet) to view the required examination joint; this may be particularly relevant with elderly patients or those with a disability, e.g., visual impairment.

The requirement for appropriate attire to allow visual access for remote examination is vital [26]. Patients should be advised to be mindful of this when dressing for their consultation - outlined in Table 2.

Technical Set-Up

While online platforms may vary between practices (Appendix 1.1), it is strongly recommended that information detailing the function of the chosen software is sent to patients in advance. This should include information on how to access the teleconsultation. It is essential that this information is clear and coherent perhaps in the form of a pictorial diagram or a link to an explanatory video; the technical set-up can have a significant effect on the accuracy of teleconsultation [27].

The creation of a frequently asked question list is also recommended [25]. Where there is a personalised link for the patient’s teleconsultation, it is important that this link is sent to the patient’s device being used for the consultation, e.g., send the link via email, text or both. With elderly patients, third-party input may be helpful, so consider sending the link to a friend or relative (with the appropriate consent in place) if the patient does not have an appropriate internet connection. Similarly, a third party may also be beneficial where visualisation is needed from an awkward angle or for taking photographs - as has been found with dermatological telemedicine.

Share Screen Function

A share screen function is a tool that allows the meeting host to present a document or image so that others can view it. This could be useful in a primary care setting using pre-prepared photos or videos [26,28], e.g., to show patients how to perform MSK tests which may otherwise be complicated to describe. Examination photos that may be helpful to demonstrate this for patients are discussed later under remote MSK examination.

Contingency Plan

Although technological developments are rapid, there is still a potential risk of technical failure. Therefore, having a plan to manage this is recommended, such as changing to an audio consultation via telephone [13]. At the start of the teleconsultation, this contingency plan should be agreed upon.

Patient Teleconsultation Advice Sheet

An information sheet for patients, summarising important information, which could be sent to patients in advance of their consultation, can be found in Table 2.

Advice for the Practitioner

As a practitioner, it is important to ensure that the video consultation still feels personal for the patient. Methods such as a ‘virtual handshake’ and making eye contact with the camera whilst introducing yourself help to strengthen the doctor-patient relationship. It is also important to recognise the potential for audio delays, so giving time for the patient to reply is vital. Pacing cues such as gestures can also be useful for this. A number of authors also recognise the need to summarise the consultation at the end; this gives the patient the opportunity to ask questions that can be difficult to ask during the consultation [24,29,30] (Appendix 1.4 and 1.5).

It is advised that health care providers wear smart or professional attire. As with patients, plain background with adequate front lighting is essential (Appendix 1.3). It is also recommended to sit about 2-feet (60 cm) away from the camera, placing yourself centrally to the screen. Gestures that are commonplace in a face-to-face consultation, for example, leaning in, can reduce the clarity on screen due to changes in focus. The clinician should try and maintain a constant 2-feet (60 cm) distance from the camera.

With the new implementation of face masks in face-to-face consultations, the remote assessment may allow practitioners to be more perceptive to any patient reactions which may be hidden by a mask. Thus, there is a possibility that teleconsultation may become the preferred option for assessment in the future if the requirement for face-coverings continues.

MSK remote examination framework

**Figure 3:**
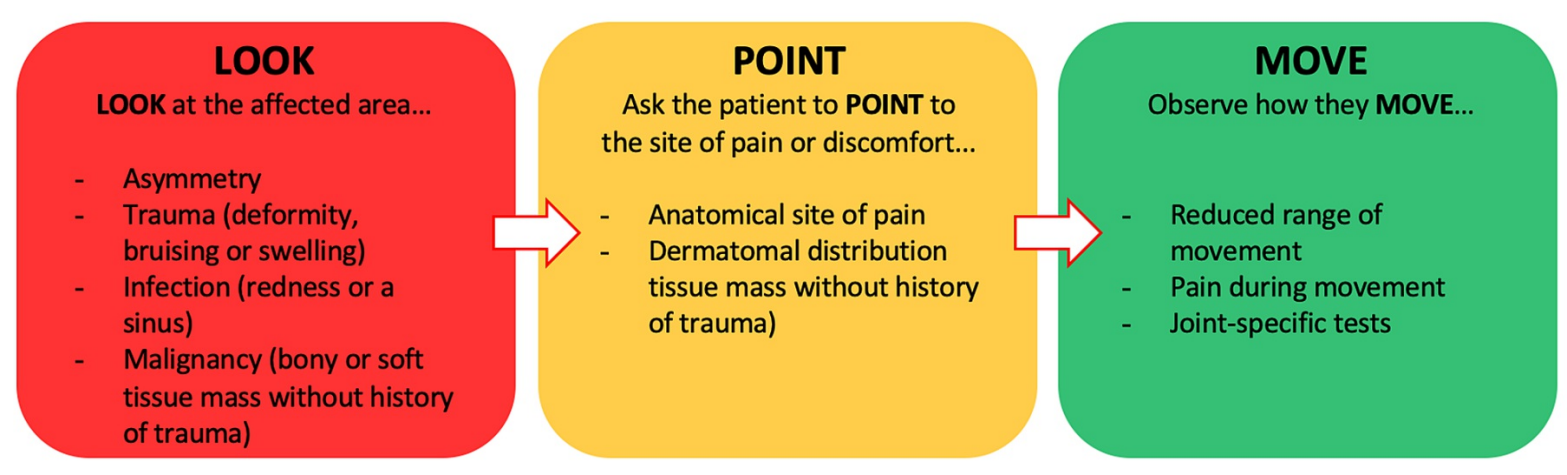
Look, Point, Move

With the implementation of a framework, it is less likely the clinician will miss pathology during teleconsultation. Thus, we have adapted the traditional triad of ‘look, feel, move’ to ‘look, point, move’ for remote MSK examination (Figure 3). This can be distilled to LOOK at the affected area, ask the patient to POINT to the site of any pain and then observe how the patient MOVEs.

**Figure 4:**
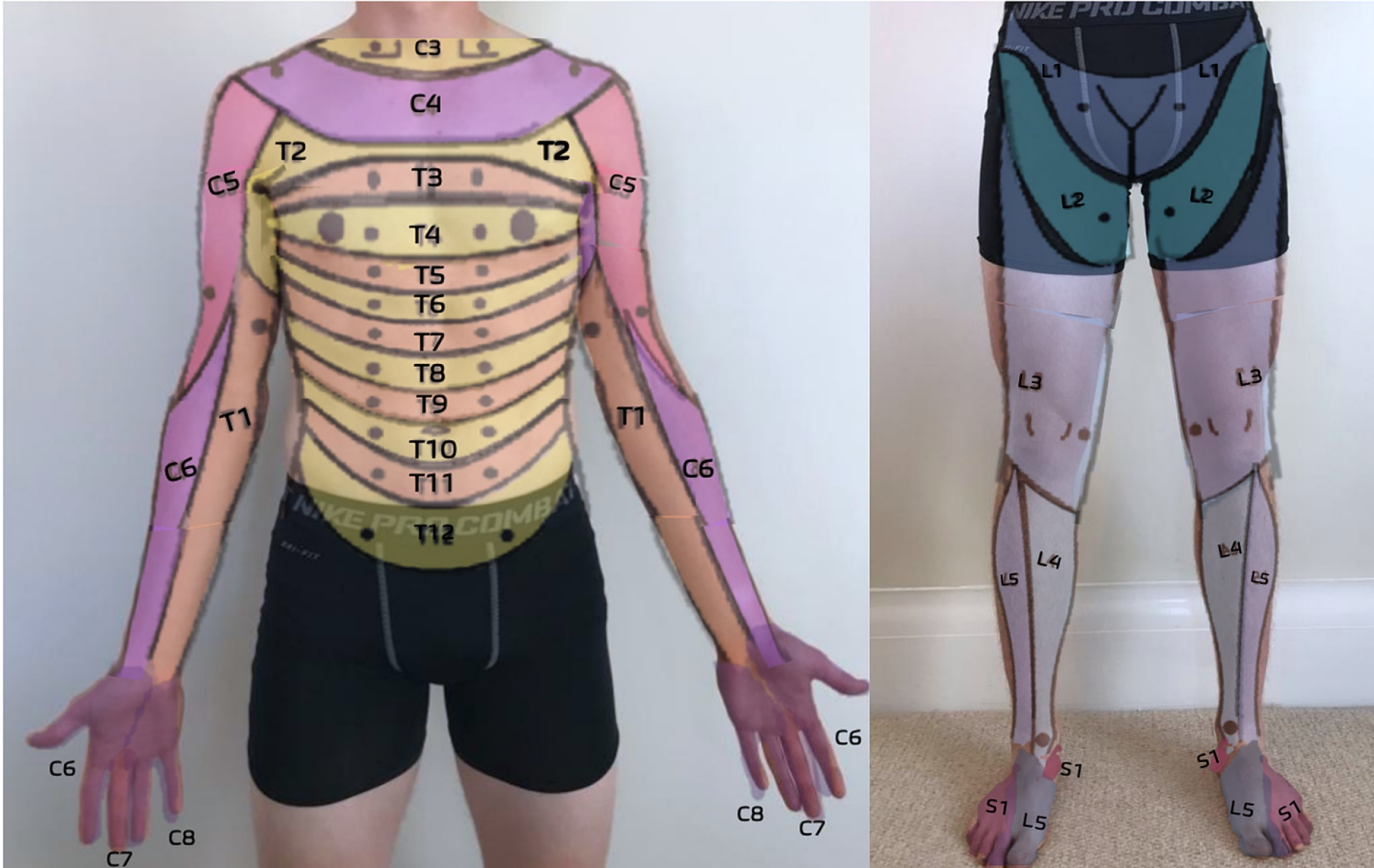
Dermatome Reference for Primary Care Musculoskeletal Medicine

A dermatome map is included in Figure 4.

Instructions for specific joint examinations and example images can be found in Figures 5-17 and are intended as a clinician resource. Normal tests are shown in green, with abnormal tests superimposed in red where applicable.

**Figure 5:**
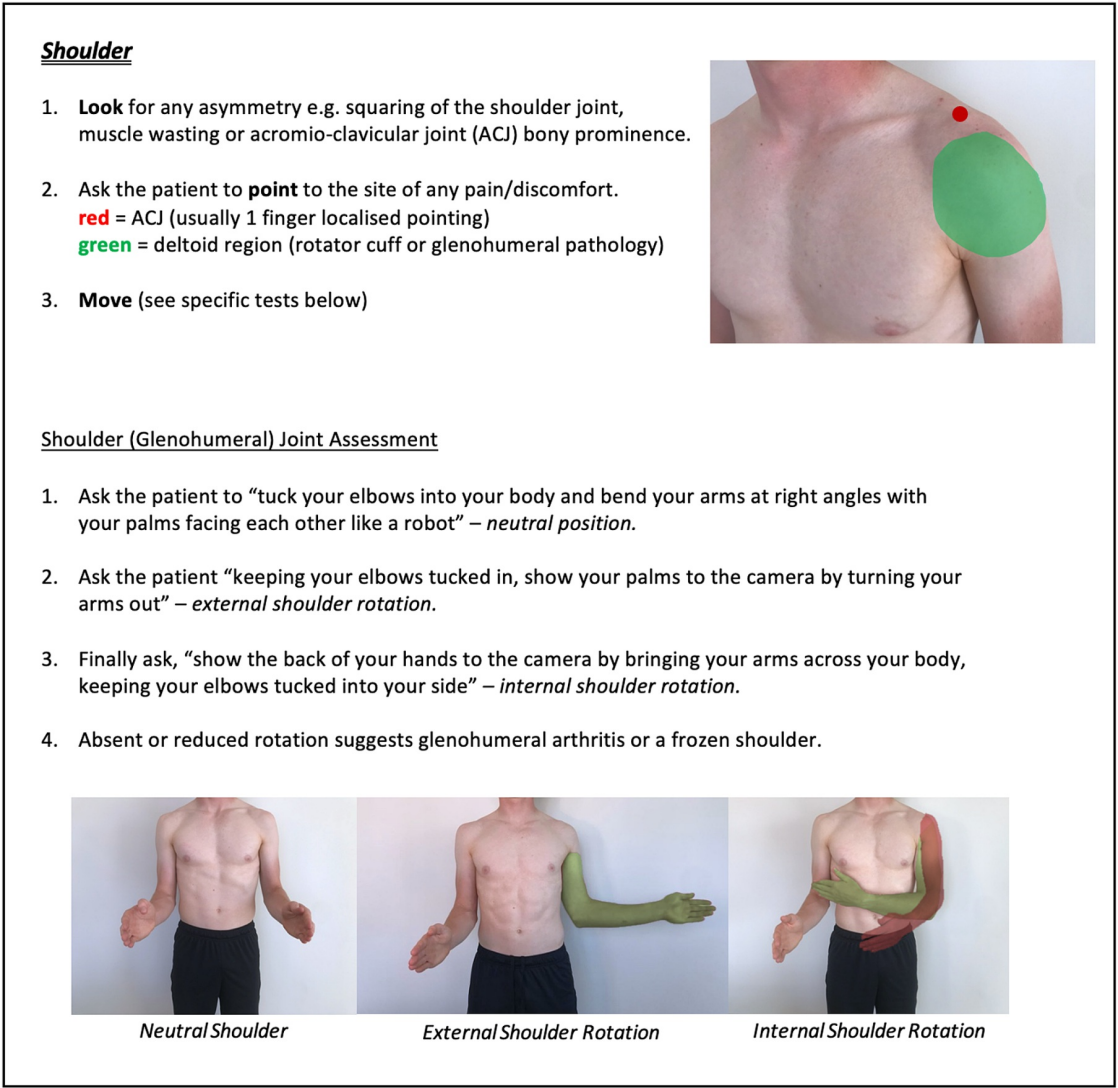
Shoulder (Global) Clinician Resource

**Figure 6:**
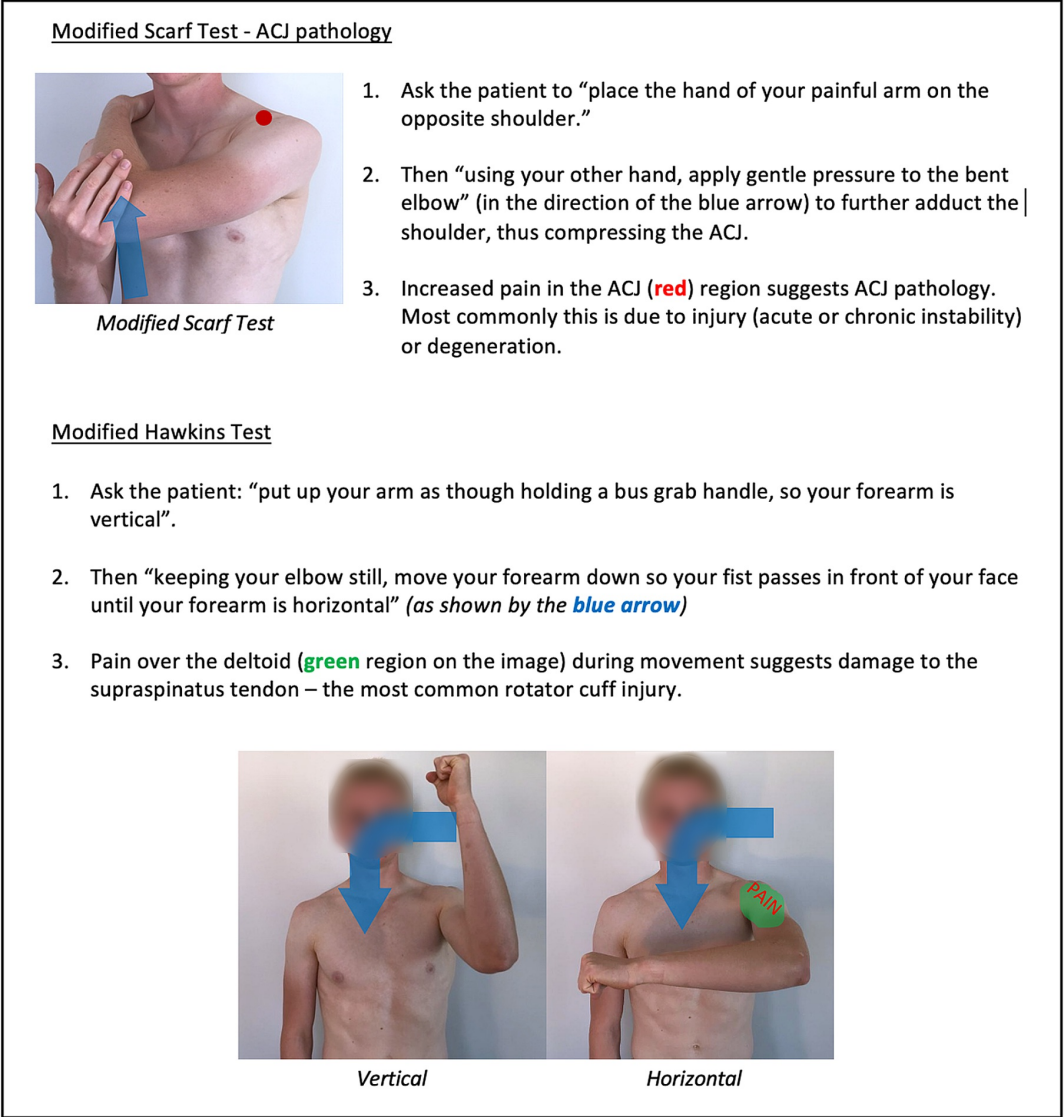
Shoulder (Specific) Clinician Resource

**Figure 7:**
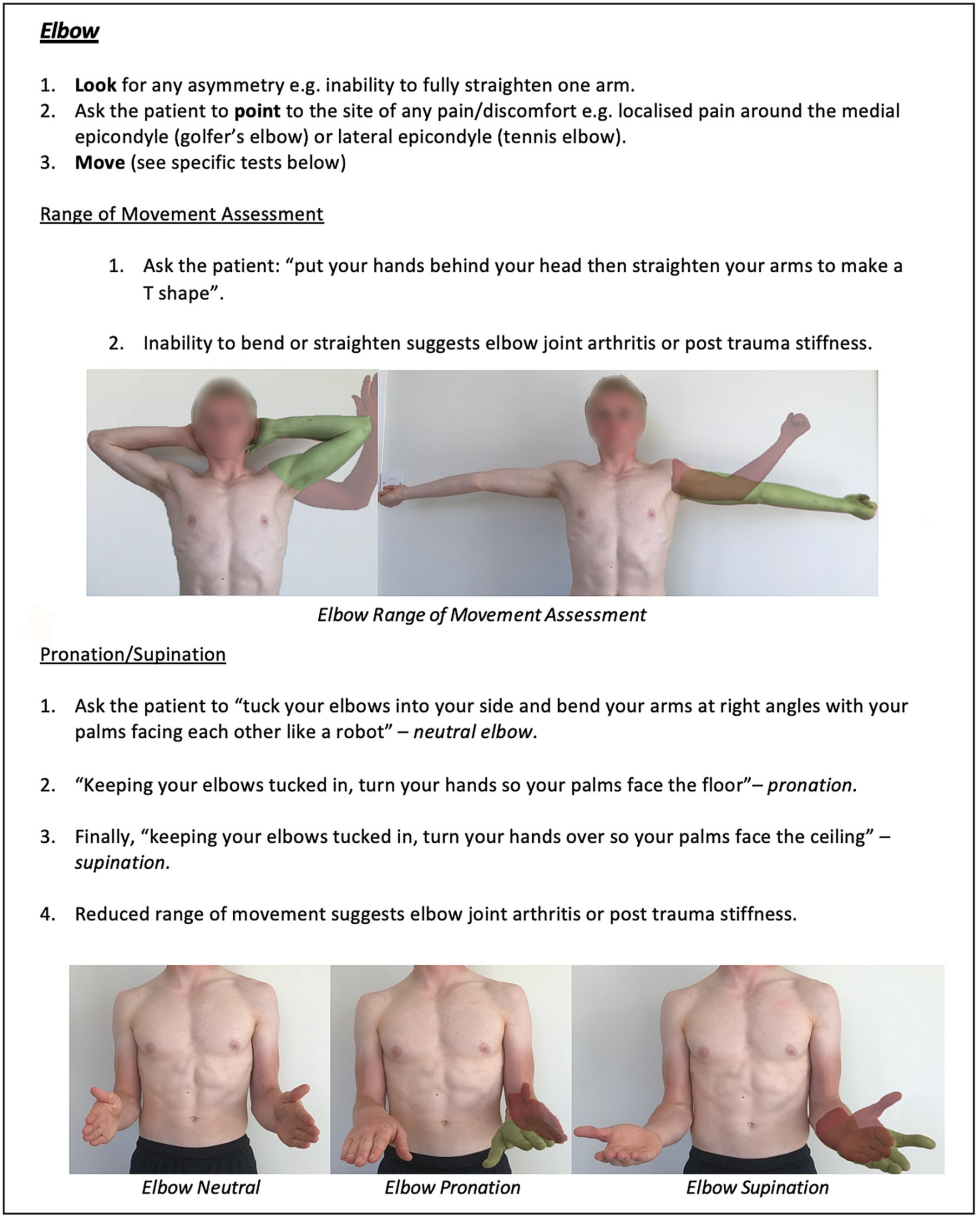
Elbow Clinician Resource

**Figure 8:**
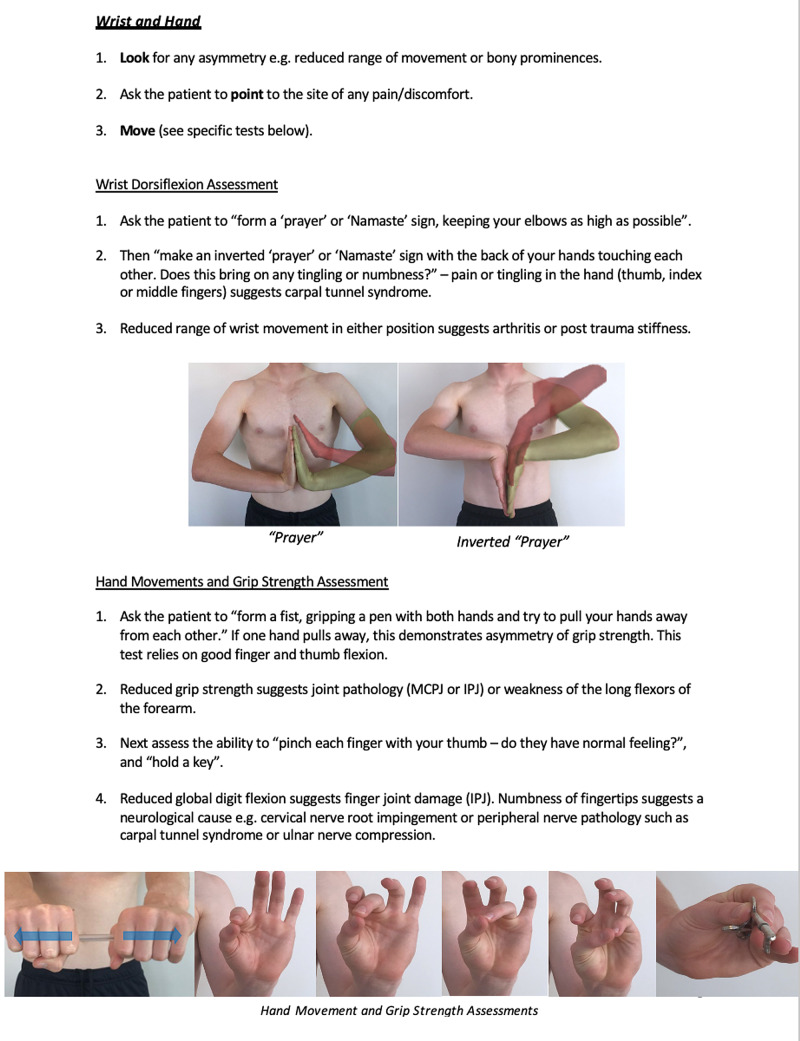
Wrist and Hand Clinician Resource

**Figure 9:**
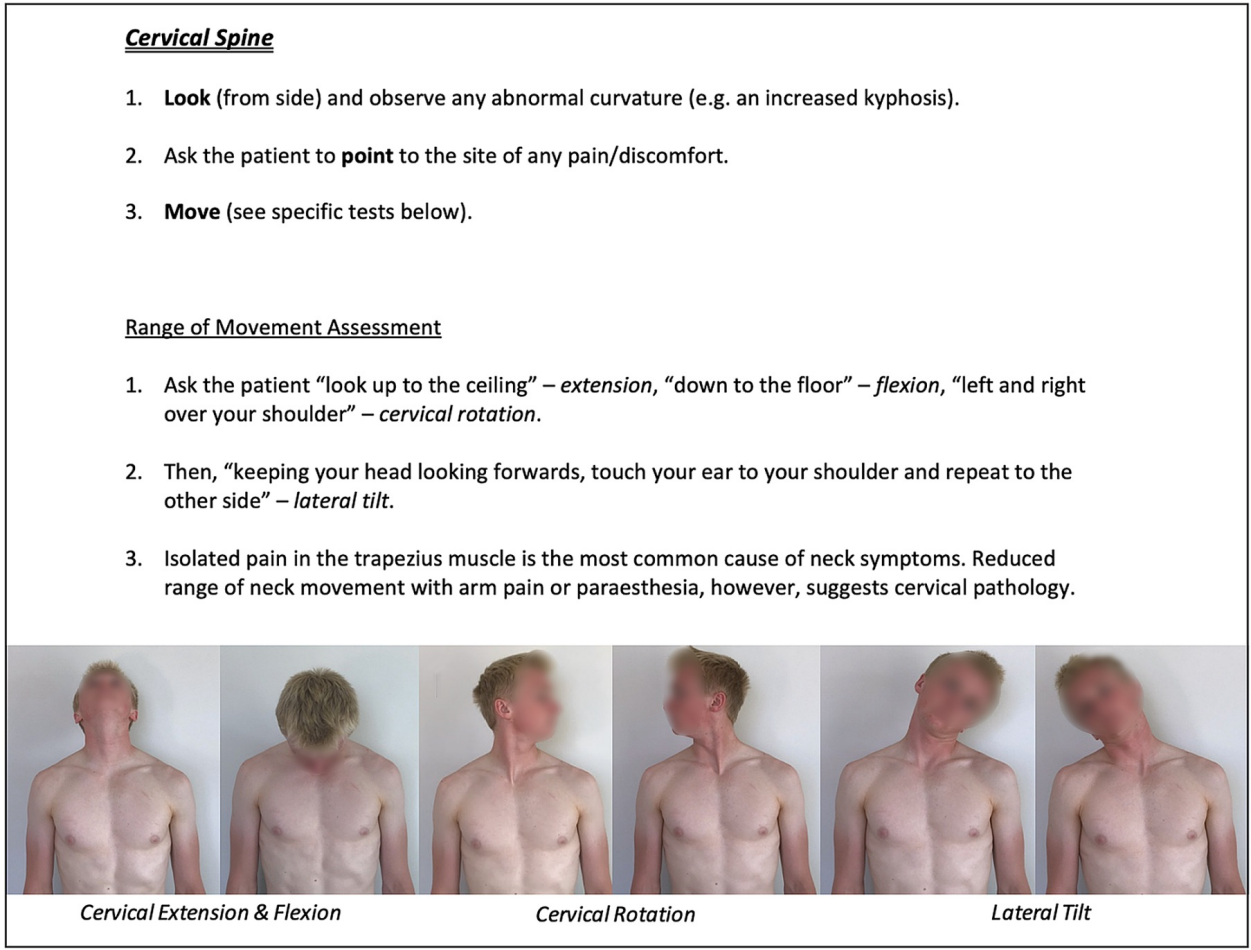
Cervical Spine Clinician Resource

**Figure 10:**
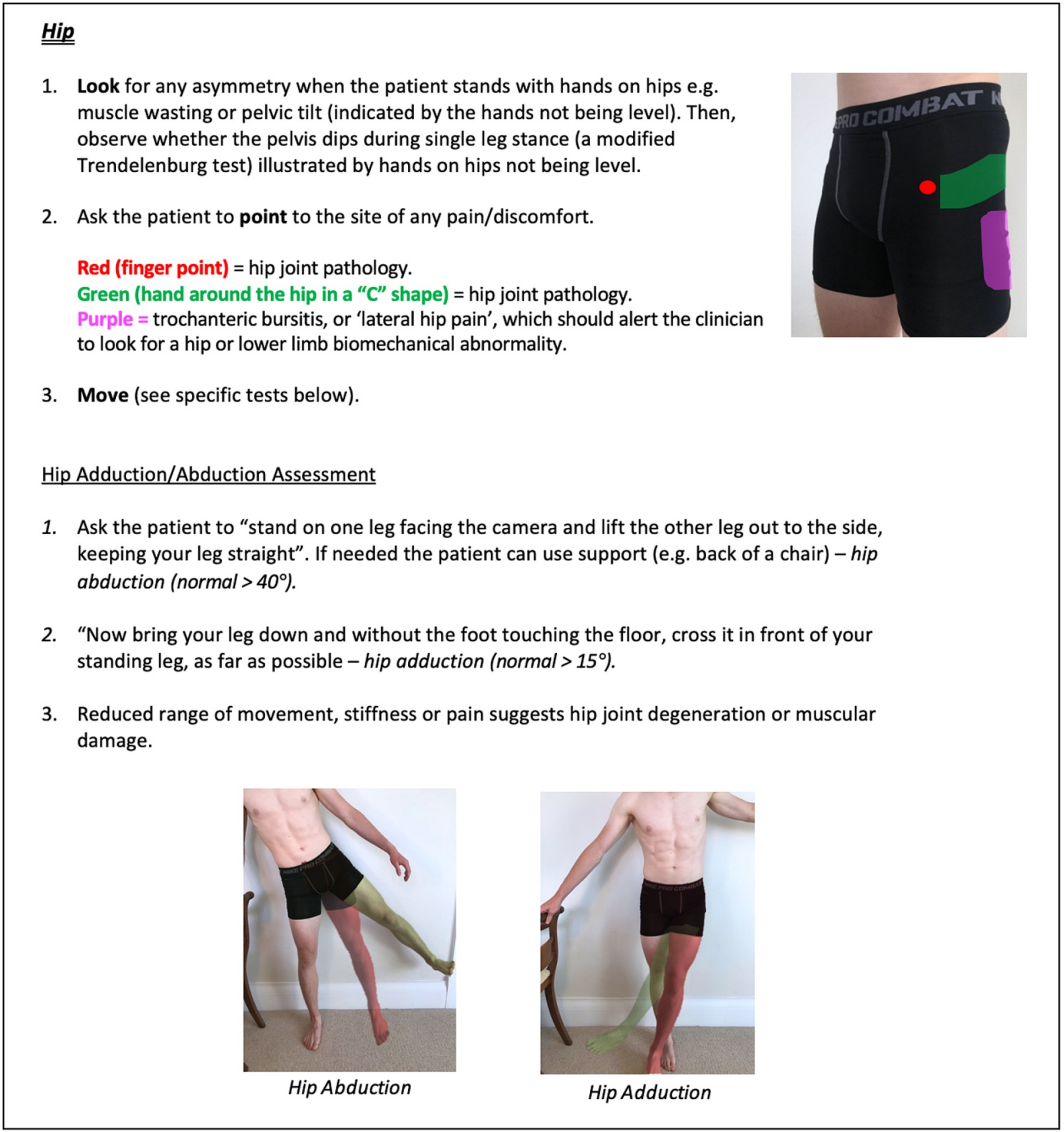
Hip Clinician Resource (Abduction/Adduction Assessment)

**Figure 11:**
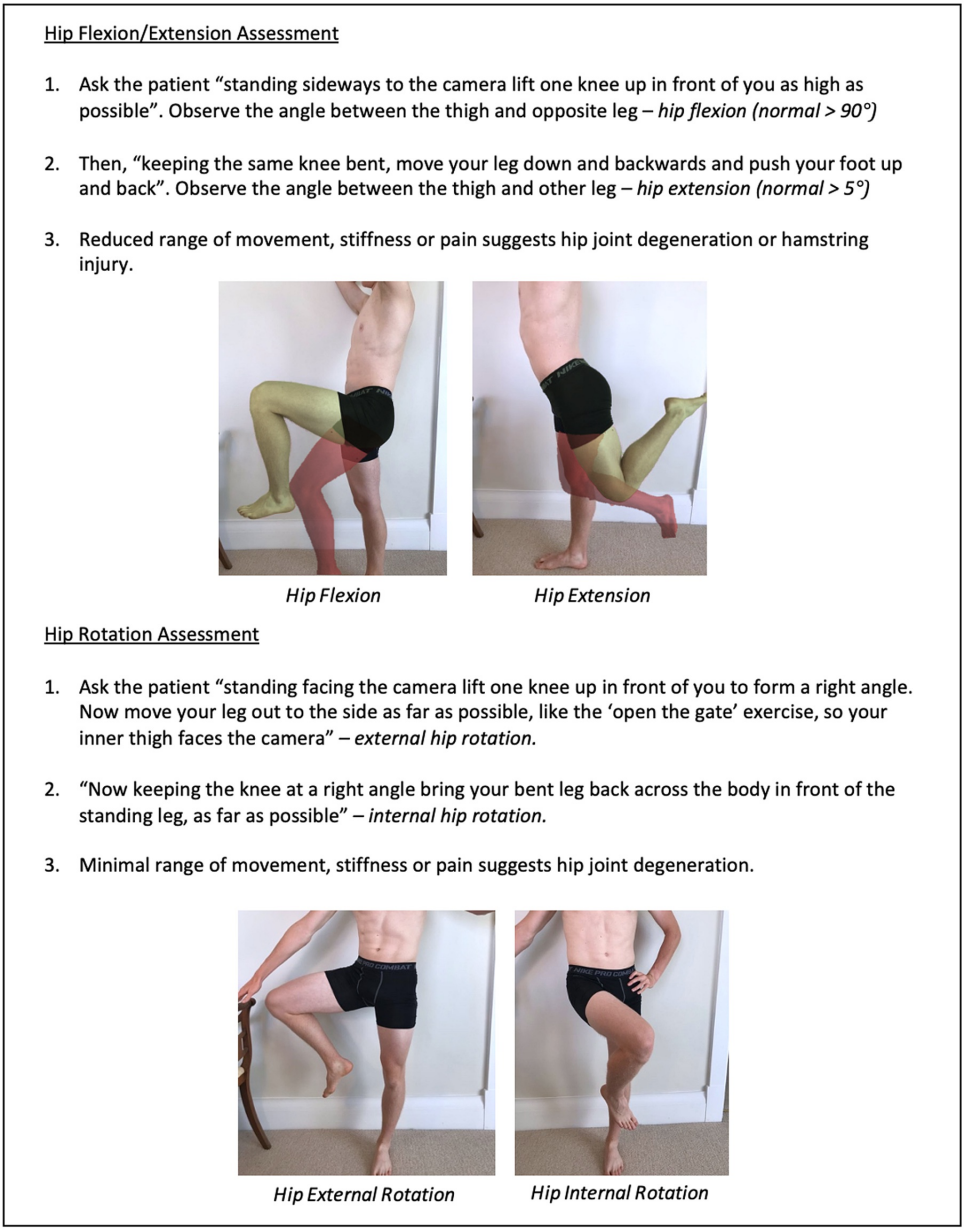
Hip Clinician Resource (Flexion/Extension and Rotation Assessment)

**Figure 12:**
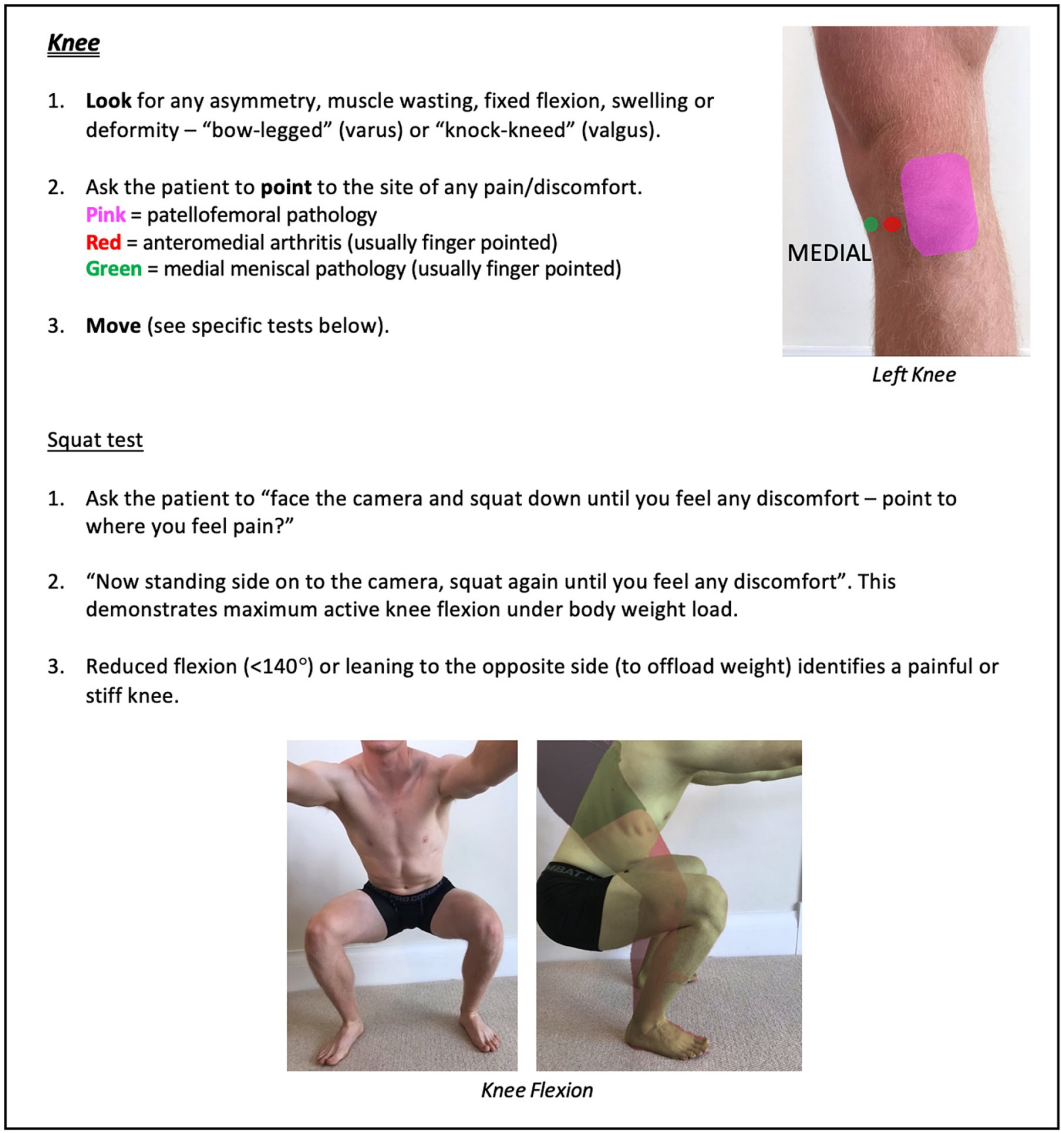
Knee Clinician Resource (Squat Test)

**Figure 13:**
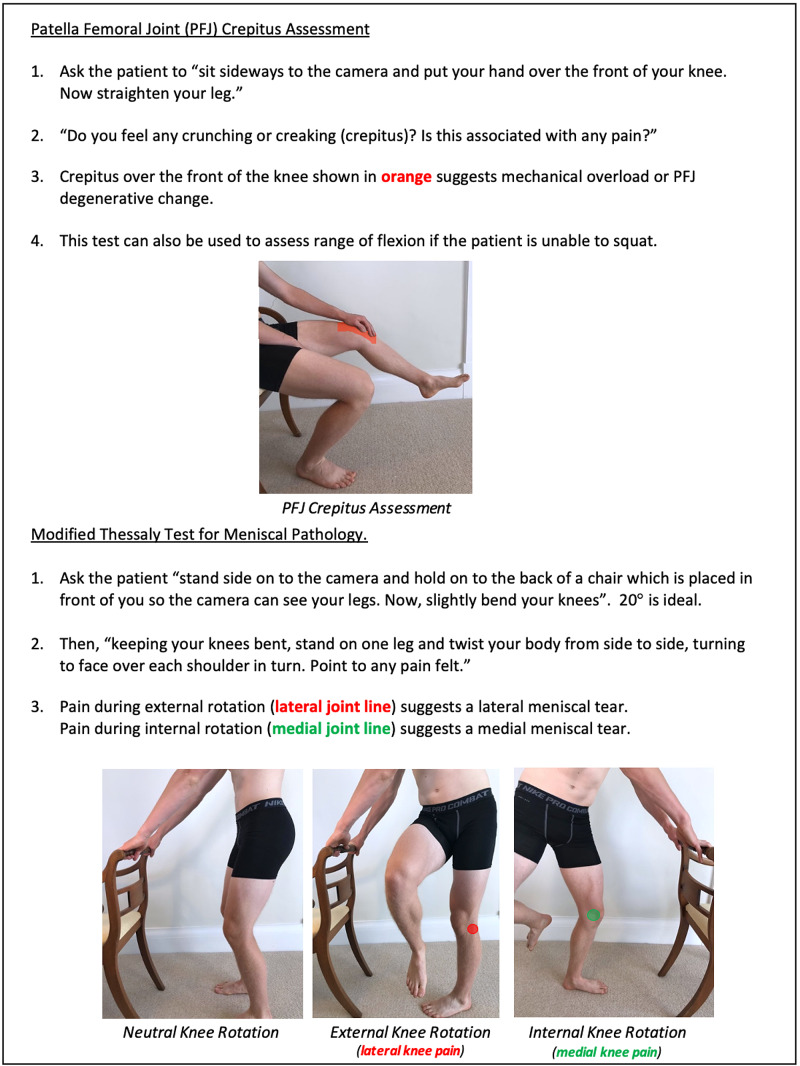
Knee Clinician Resource (PFJ Crepitus Assessment and Modified Thessaly Test) PFJ: patella-femoral joint.

**Figure 14:**
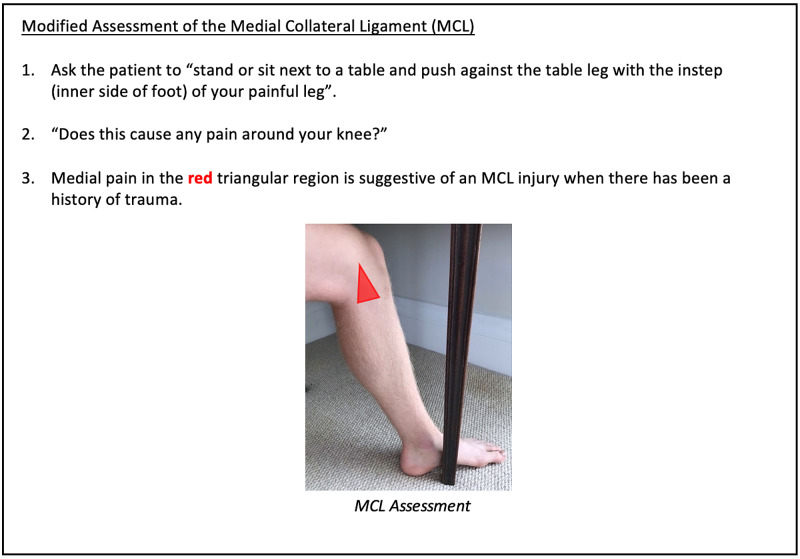
Knee Clinician Resource (Modified Assessment of MCL) MCL: medial collateral ligament.

**Figure 15:**
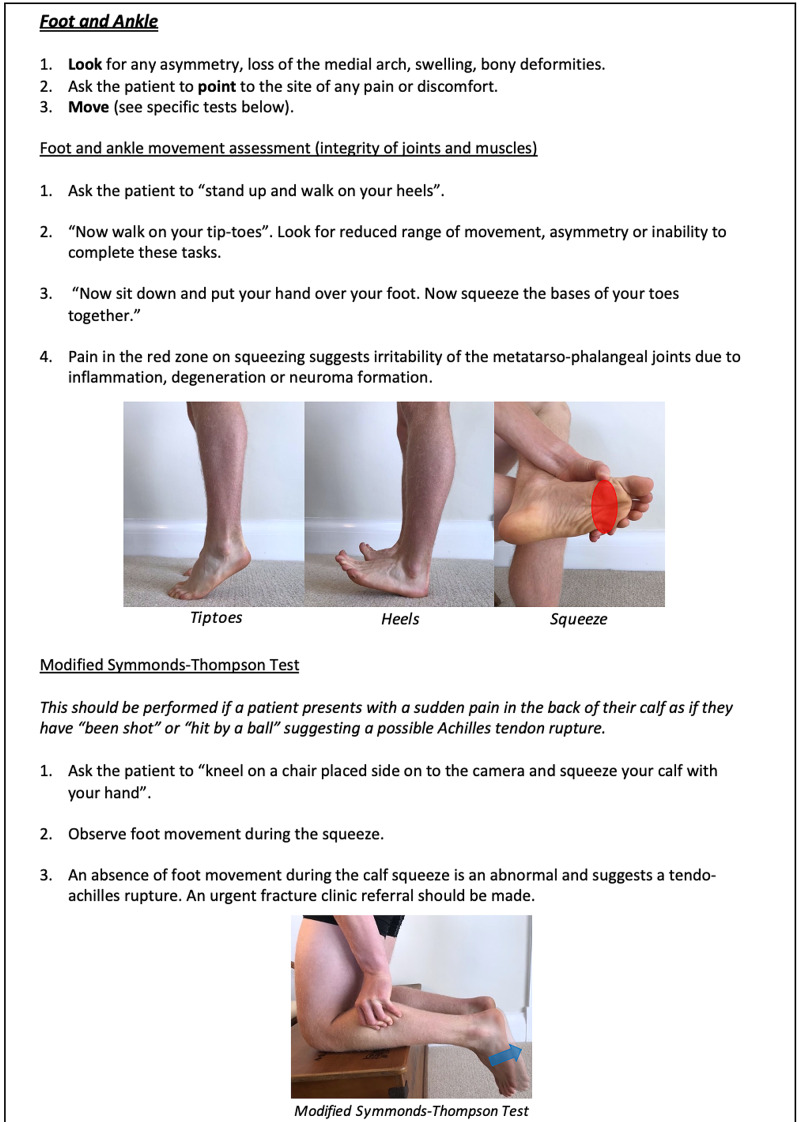
Foot and Ankle Clinician Resource

**Figure 16:**
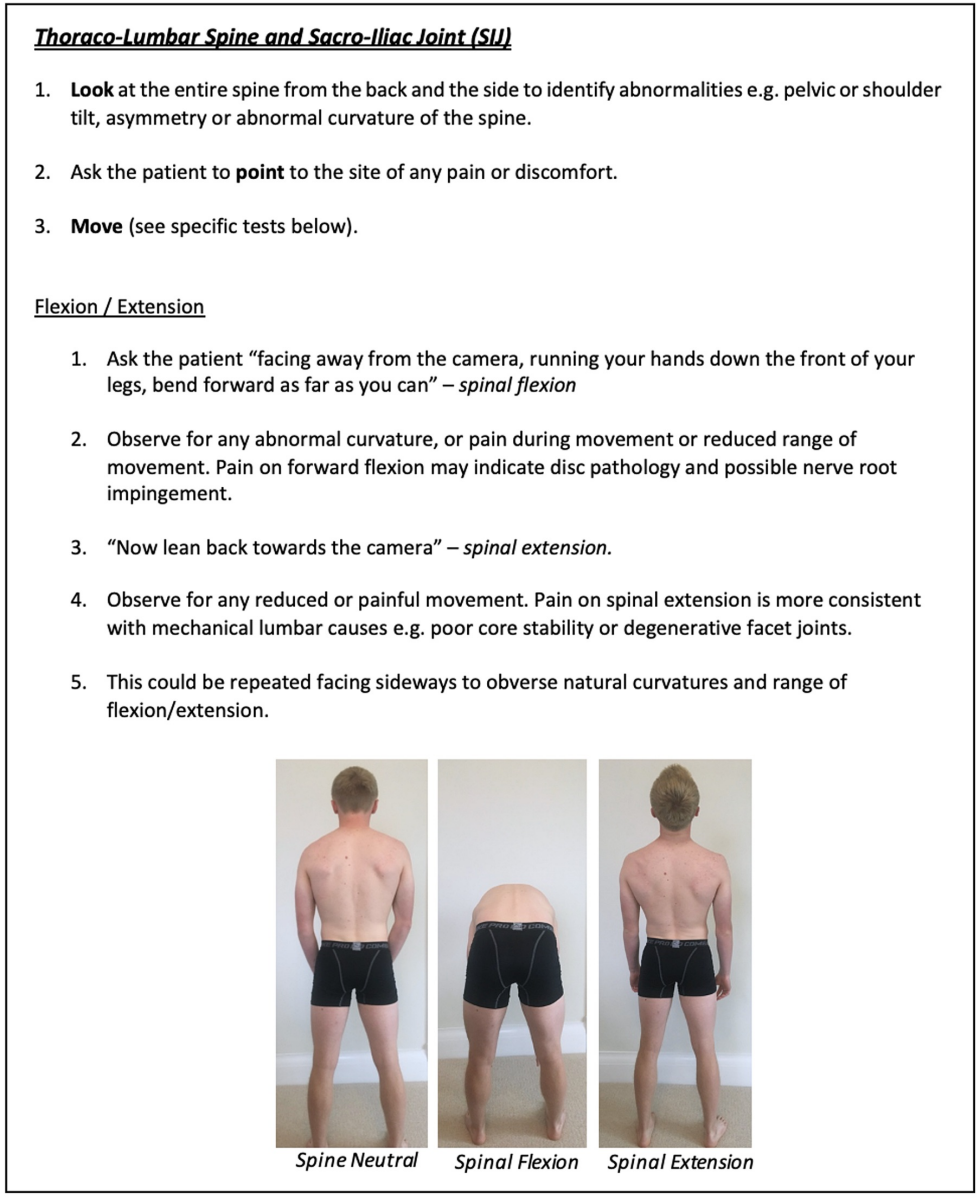
Thoraco-Lumbar Spine and SIJ Clinician Resource Showing Flexion/Extension Assessment SIJ: sacro-iliac joint.

**Figure 17:**
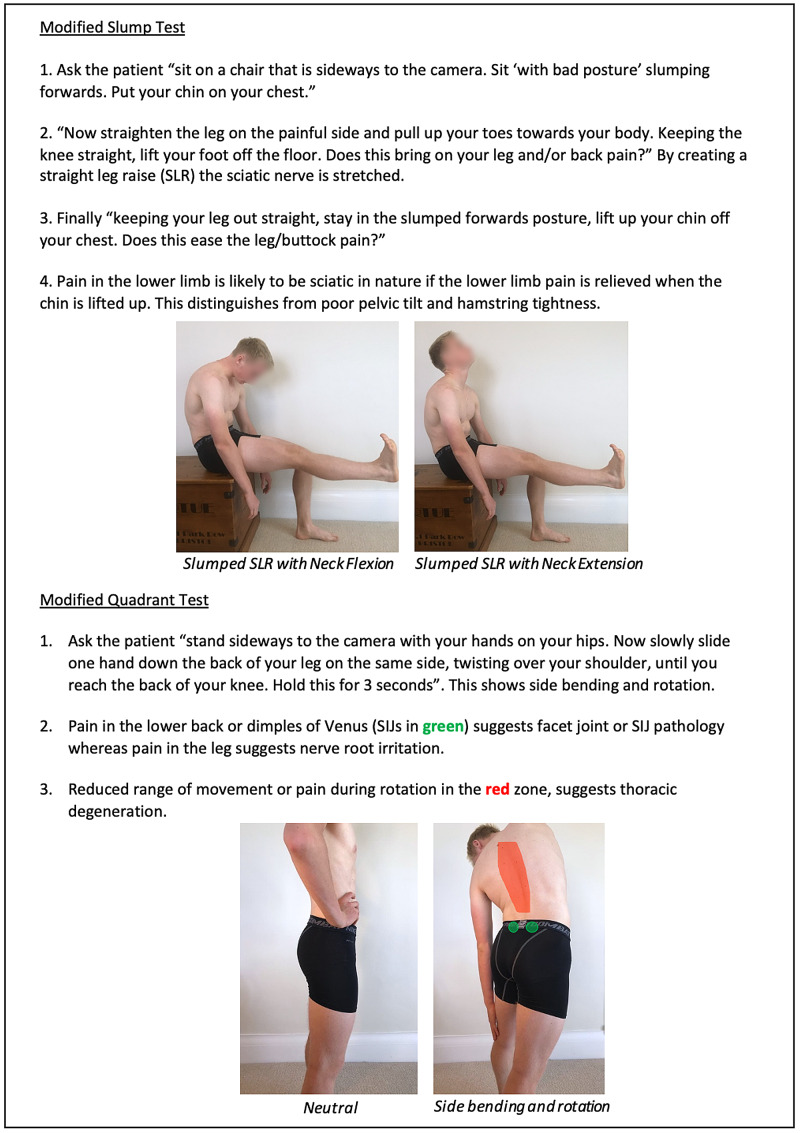
Thoraco-Lumbar Spine and SIJ Clinician Resource Showing Modified Slump and Quadrant Test

**Figure 18:**
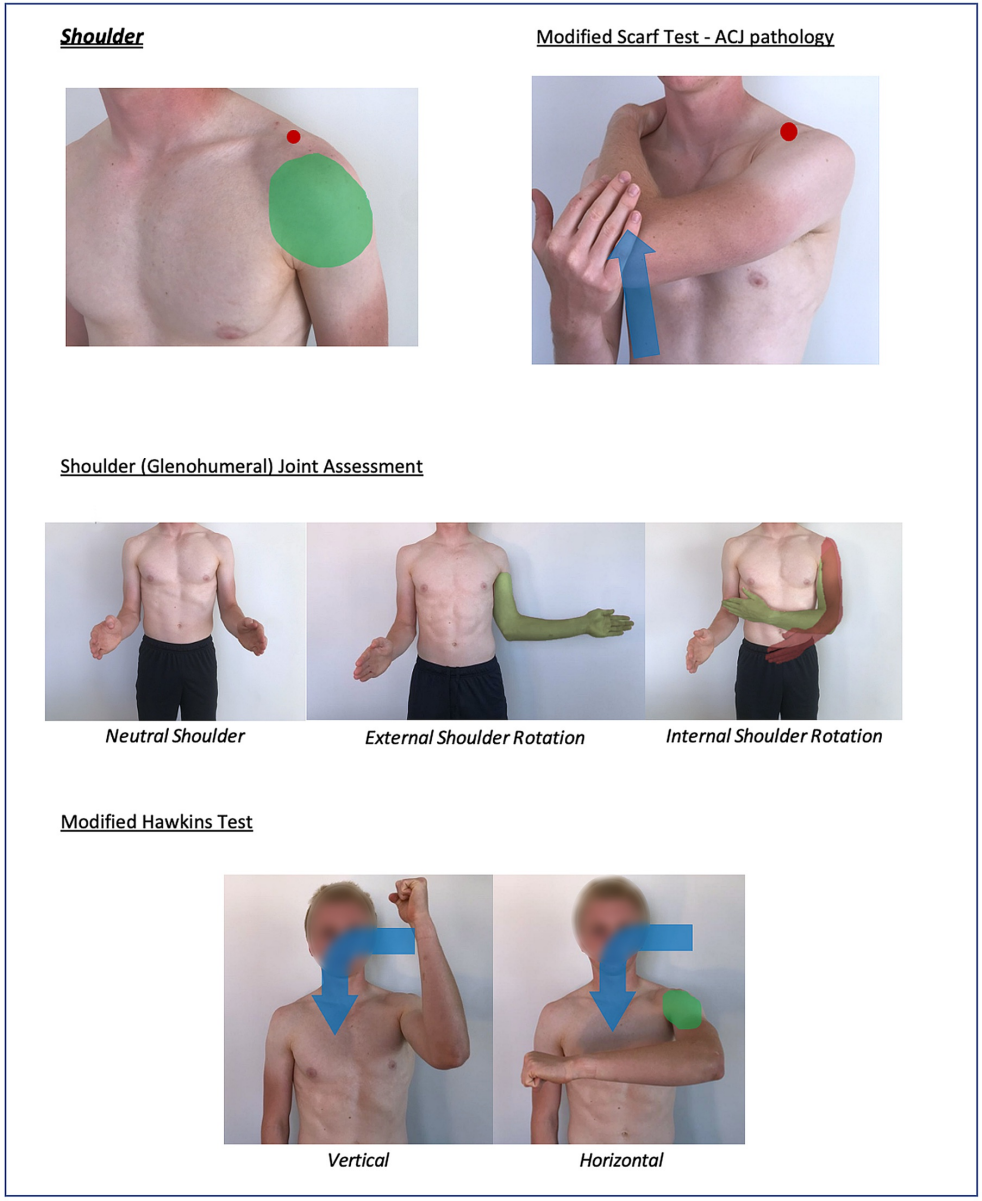
Shoulder Patient Resource

**Figure 19:**
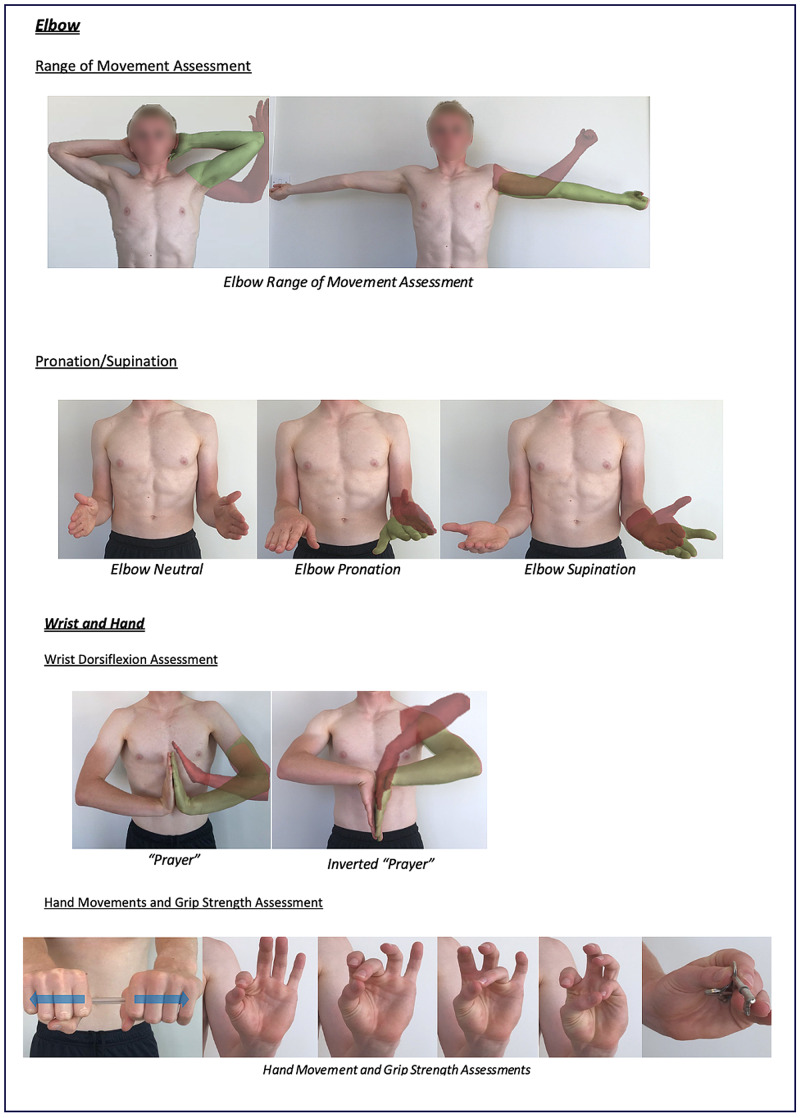
Elbow, Wrist and Hand Patient Resource

**Figure 20:**
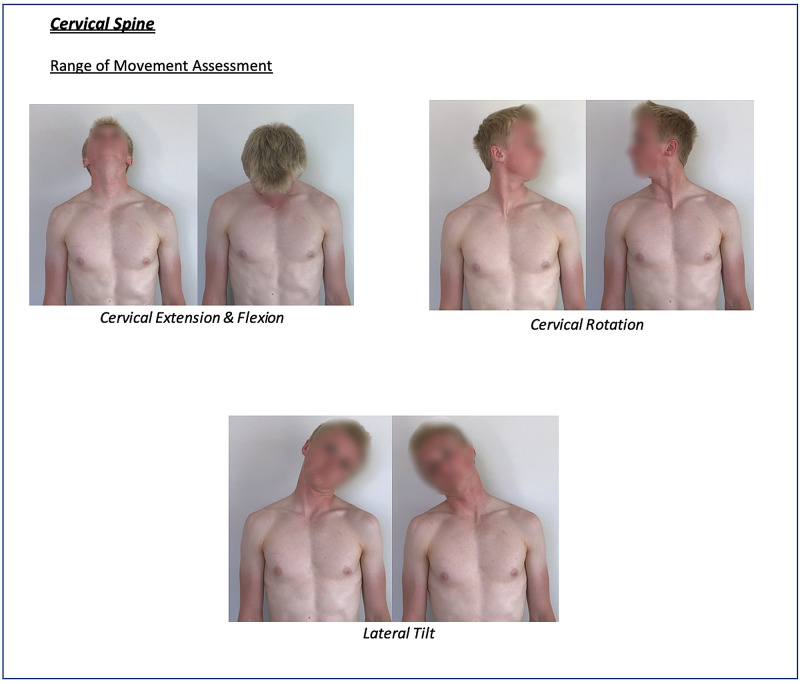
Cervical Spine Patient Resource

**Figure 21:**
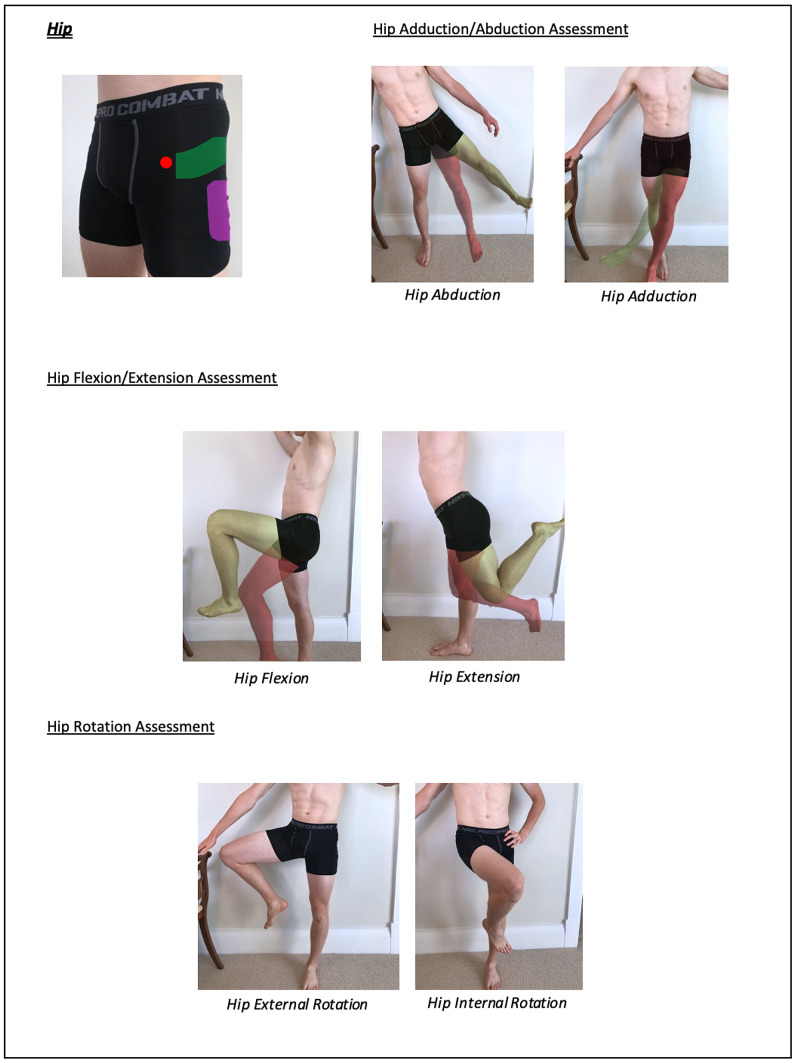
Hip Patient Resource

**Figure 22:**
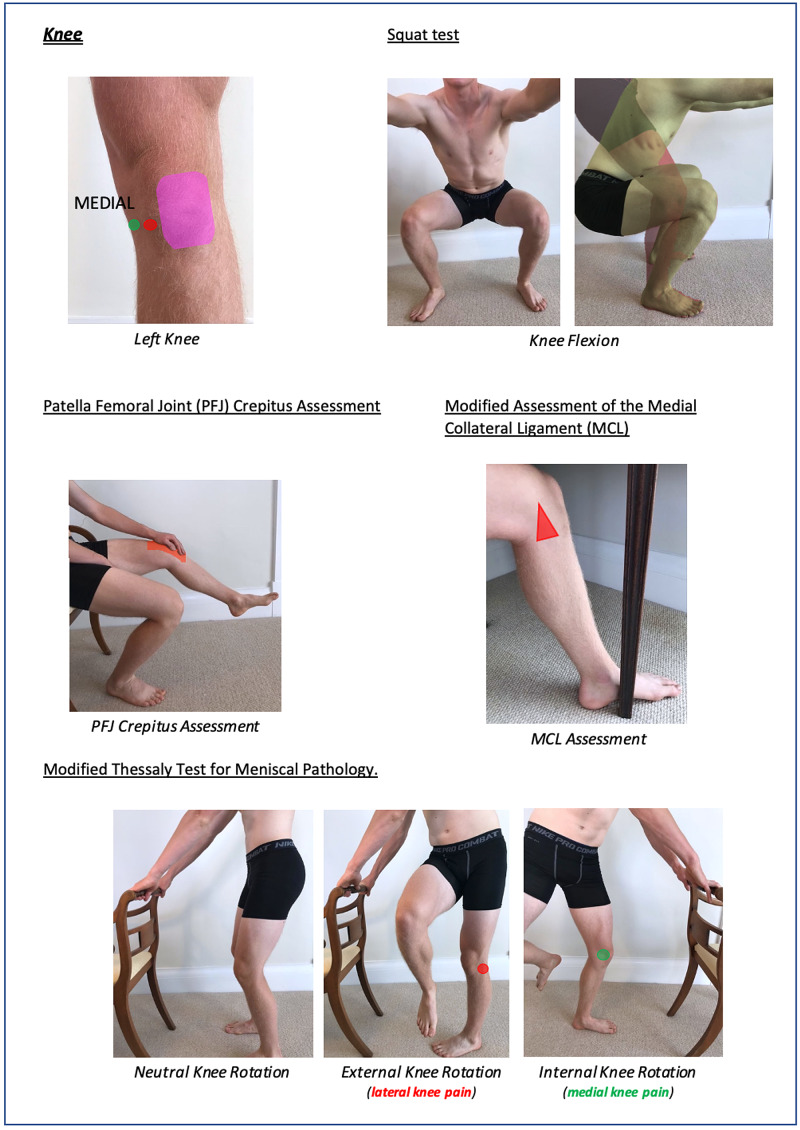
Knee Patient Resource

**Figure 23:**
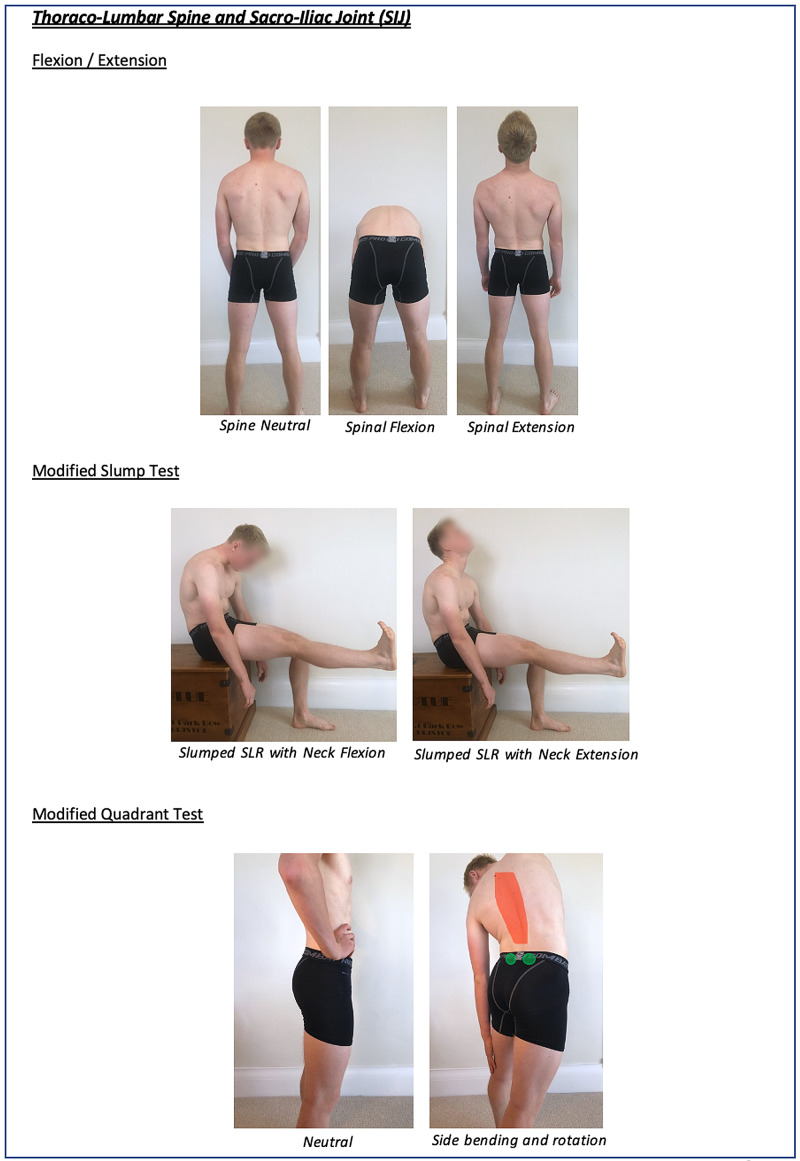
Thoraco-Lumbar Spine and SIJ Patient Resource SIJ: sacro-iliac joint.

Corresponding images of tests for demonstration purposes (e.g., with a share screen function) to show the specific movements can be found in Figures 18-23.

## Discussion

Summary

This is a systematic literature review of MSK telemedicine from which practical advice and evidence-based MSK tests have been developed. There is currently no published evidence relating to MSK remote consultation in a primary care setting. However, there is evidence to support the use of MSK telemedicine in secondary care and physiotherapy practice; it seems reasonable to extrapolate from this, some techniques suitable for use in a primary care setting. Where there is a lack of such evidence, modified traditional tests are suggested to allow a complete framework for remote MSK examination - using a system approach of ‘look, point, move’ followed by modified special tests, for use in a primary care setting. 

Strengths and limitations

In addition to a standard literature review, websites (e.g., Google and YouTube) and education websites were also targeted. There is strong evidence for the use of teleconsultation in MSK physiotherapy and secondary care practice. The gaps in this evidence have been filled by the experience of a practicing GP and orthopaedic surgeon.

The primary limitation is the lack of validation for these remote MSK examinations performed in a primary care setting. Whilst assessments of the modified examinations were conducted to check the efficacy of the suggested verbal instructions, further research must be performed to compare teleconsultation with face-to-face MSK examinations to establish validity and diagnostic reliability.

Comparison with existing literature

Whilst there is a lack of published evidence regarding primary care MSK teleconsultation, there is evidence from physiotherapy and orthopaedic literature to suggest the efficacy of remote consultation in MSK conditions; this highlights the demand and benefit of MSK telemedicine in non-primary care settings. The current MSK telemedicine literature demonstrates its validity, reliability, diagnostic accuracy and patient satisfaction in other settings that are likely to transfer into primary care. 

Implications for research and practice

Using this MSK examination framework, primary care practices should be able to conduct MSK video consultation more effectively and efficiently. This may be achieved by downloading the above figures where there is a clinician (Figures 2-14) and patient (Figures 15-21) information. Table 3 offers a pre-consultation information sheet ideal to send to patients prior to the e-consult, which should enhance the efficiency of a consultation. In the clinician resource (Figures 2-14), the images of specific examinations are partnered with clear suggested verbal instructions for clinician use. The corresponding patient resource (Figures 15-21) may be helpful to demonstrate examinations to patients, using a share screen function or by prior sending.

This framework could also be used as a reference resource for students when learning MSK examination in both remote and face-to-face environments.

Abnormal findings should prompt the clinician’s standard management. This may involve an additional face-to-face examination, by the same clinician or another GP with more MSK experience, a physiotherapist, the local MSK triage service or a secondary care MSK specialist; the exact referral pathway will depend on the local healthcare system.

## Conclusions

With 21% of primary care consultations relating to MSK conditions and limited means of performing face-to-face MSK examination due to COVID-19, there needs to be a recognised framework for assessing the MSK system remotely. To the best of our knowledge, this evidence does not exist for primary care remote MSK examination. This paper demonstrates evidence-based practical advice (from non-primary care settings) and a proposed modified MSK remote examination framework for use in a primary care setting.

## Appendices

Additional information sources

1.1     NHS Advice on using video consultation systems. https://digital.nhs.uk/services/gp-it-futures-systems/approved-econsultation-systems

1.2    Red Whale MSK Remote Consultation Webinar. https://www.gp-update.co.uk/webinars/OWMSK300820?utm_medium=email&utm_campaign=Pearl%20230720%20Free%20MSK%20Remote%20Assessment%20Webinar&utm_content=Pearl%20230720%20Free%20MSK%20Remote%20Assessment%20Webinar+CID_ef2e0a5082b5fc3a948dff87ae3ab54a&utm_source=Email%20marketing%20software&utm_term=Watch%20our%20FREE%20webinar%20-%20MSK%20Remote%20Assessment%20Guide

1.3    The Telemedicine Musculoskeletal Examination (video) - Mayo Clinic - YouTube. https://www.youtube.com/watch?v=U3AswzGgDS8&feature=share

1.4    Roger Neighbour. Top 10 tips for successful GP video consultations. RCGP online. https://www.rcgp.org.uk/about-us/rcgp-blog/top-10-tips-for-successful-gp-video-consultations.aspx

1.5    COVID-19: video consultations and homeworking - BMA online https://www.bma.org.uk/advice-and-support/covid-19/adapting-to-covid/covid-19-video-consultations-and-homeworking
